# Therapeutic evaluation of magnetic hyperthermia using Fe_3_O_4_-aminosilane-coated iron oxide nanoparticles in glioblastoma animal model

**DOI:** 10.31744/einstein_journal/2019AO4786

**Published:** 2019-07-25

**Authors:** Gabriel Nery de Albuquerque Rego, Javier Bustamante Mamani, Taylla Klei Felix Souza, Mariana Penteado Nucci, Helio Rodrigues da Silva, Lionel Fernel Gamarra

**Affiliations:** 1 Hospital Israelita Albert Einstein, São Paulo, SP, Brazil; 2 Hospital das Clínicas, Faculdade de Medicina, Universidade de São Paulo, São Paulo, SP, Brazil

**Keywords:** Glioblastoma/therapy, Magnetic, hyperthermia, Nanoparticles, Magnetite nanoparticles, Cell tracking/methods, Aminosilane, Tumoral model, Rats, Glioblastoma/terapia, Magneto, hipertermia, Nanopartículas, Nanopartículas de magnetita, Aminosilana, Modelo tumoral, Ratos

## Abstract

**Objective:**

To evaluate the potential of magnetic hyperthermia using aminosilane-coated superparamagnetic iron oxide nanoparticles in glioblastoma tumor model.

**Methods:**

The aminosilane-coated superparamagnetic iron oxide nanoparticles were analyzed as to their stability in aqueous medium and their heating potential through specific absorption rate, when submitted to magnetic hyperthermia with different frequencies and intensities of alternating magnetic field. In magnetic hyperthermia *in vitro* assays, the C6 cells cultured and transduced with luciferase were analyzed by bioluminescence in the absence/presence of alternating magnetic field, and also with and without aminosilane-coated superparamagnetic iron oxide nanoparticles. In the *in vivo* study, the measurement of bioluminescence was performed 21 days after glioblastoma induction with C6 cells in rats. After 24 hours, the aminosilane-coated superparamagnetic iron oxide nanoparticles were implanted in animals, and magnetic hyperthermia was performed for 40 minutes, using the best conditions of frequency and intensity of alternating magnetic field tested in the *in vitro* study (the highest specific absorption rate value) and verified the difference of bioluminescence before and after magnetic hyperthermia.

**Results:**

The aminosilane-coated superparamagnetic iron oxide nanoparticles were stable, and their heating capacity increased along with higher frequency and intensity of alternating magnetic field. The magnetic hyperthermia application with 874kHz and 200 Gauss of alternating magnetic field determined the best value of specific absorption rate (194.917W/g). When these magnetic hyperthermia parameters were used in *in vitro* and *in vivo* analysis, resulted in cell death of 52.0% and 32.8%, respectively, detected by bioluminescence.

**Conclusion:**

The magnetic hyperthermia was promissing for the therapeutical process of glioblastoma tumors in animal model, using aminosilane-coated superparamagnetic iron oxide nanoparticles, which presented high specific absorption rate.

## RESUMO

**Objetivo:**

Avaliar o potencial da técnica de magneto-hipertermia utilizando nanopartículas superparamagnéticas de óxido de ferro recobertas com aminosilana em modelo de tumores de glioblastoma.

**Métodos:**

As nanopartículas superparamagnéticas de óxido de ferro recobertas com aminosilana foram avaliadas quanto à sua estabilidade em meio aquoso e a seu potencial de aquecimento pela taxa de absorção específica, quando submetidas à magneto-hipertermia, com diferentes frequências e intensidades de campo magnético alternado. Nos ensaios de magneto-hipertermia *in vitro*, as células C6 cultivadas e transduzidas com luciferase foram avaliadas por bioluminescência na presença/ausência do campo magnético alternado, como também com e sem nanopartículas superparamagnéticas de óxido de ferro recobertas com aminosilana. No estudo *in vivo*, a medida de bioluminescência foi adquirida no 21º dia após indução do glioblastoma com células C6 nos ratos. Após 24 horas, as nanopartículas superparamagnéticas de óxido de ferro recobertas com aminosilana foram implantadas no animal, tendo sido realizada a magneto-hipertermia por 40 minutos, nas melhores condições de frequência e intensidade de campo magnético alternado testado no estudo *in vitro* (maior valor da taxa de absorção específica); foi verificada a diferença do bioluminescência antes e após a magneto-hipertermia.

**Resultados:**

As nanopartículas superparamagnéticas de óxido de ferro recobertas com aminosilana se mostraram estáveis, e sua capacidade de aquecimento aumentou com o incremento da frequência e da intensidade de campo magnético alternado. A aplicação da magneto-hipertermia, com 874kHz e 200 Gauss do campo magnético alternado, determinou o melhor valor da taxa de absorção específica (194,917W/g). Quando utilizados, estes parâmetros de magneto-hipertermia *in vitro* resultaram em morte celular de 52,0% e *in vivo* de 32,8% por bioluminescência.

**Conclusão:**

A técnica de magneto-hipertermia foi promissora para o processo terapêutico de tumores de glioblastoma no modelo animal utilizando as nanopartículas superparamagnéticas de óxido de ferro recobertas com aminosilana recobertas com aminosilana, que apresentaram alta taxa de absorção específica.

## INTRODUCTION

Glioblastoma (GBM) accounts for 47.7% of all malignant brain tumors, and only 5.6% of patients affected survive up to 5 years after diagnosis, as per the statistical report of the Central Brain Tumor Registry of the United States (CBTRUS),^(^ ^1^ ^)^ with a prognosis that is still guarded. Glioblastoma represents one of the most devastating diseases of the central nervous system due to its extremely aggressive behavior, and no satisfactory responses to the varied therapeutic modalities so far.^(^ ^2^ ^)^

Preclinical studies in GBM tumors allowed understanding and evaluation of the different strategies for the therapeutic process.^(^ ^3^ ^,^ ^4^ ^)^ Among the preclinical models of tumor induction,^(^ ^5^ ^)^ those that use C6 cells show advantages due to histological aspects and their similarities with findings in humans. These include nuclear polymorphism, high rates of mitosis, tumor necrosis foci, intratumor hemorrhage, invasion of the parenchyma, and neoangiogenesis.^(^ ^5^ ^,^ ^6^ ^)^

Since currently there is no effective treatment for GBM, alternative therapeutic mechanisms have been developed,^(^ ^7^ ^)^ among which, magnetic hyperthermia (MHT). Its relevance results from significant antitumor effects in models of GBM,^(^ ^8^ ^,^ ^9^ ^)^ as well as in other types of tumors.^(^ ^10^ ^,^ ^11^ ^)^ In the MHT technique, when directed towards the tumor and exposed to an alternating magnetic field (AMF) with appropriate intensity frequencies, the magnetic nanoparticles are heated until reaching a therapeutic temperature of 42-45°C,^(^ ^12^ ^)^ capable of affecting the tumor mass without hindering the surrounding normal tissue.^(^ ^9^ ^)^

Superparamagnetic iron oxide nanoparticles (SPIONs) have a great potential for application in the MHT, based on their magnetic properties of energy transformation, generating heat.^(^ ^9^ ^)^ Preclinical studies showed the efficacy of several biocompatible sizes and coatings of SPIONs,^(^ ^13^ ^)^ although in many of these studies the efficacy of MHT was evaluated in models of GBM tumors induced in the flanks of the animals instead of intracerebral induction.^(^ ^13^ ^)^ Applications, both of the inductive cells of the tumor as well as the SPIONs through stereotaxis, are the most adequate options for the representation of the GBM model submitted to an MHT treatment. They mimic what really happens, and due to the need to place a large quantity of SPIONs located in the tumor to increase the heating potential of thermotherapy.^(^ ^14^ ^)^

There are some aspects that still need to be assessed relative to efficacy of MHT therapy, such as the physical and chemical properties of the nanoparticles, coatings, size, type of magnetic nanoparticle, AMF parameters (frequency and intensity of oscillating field), concentration of nanoparticles, time of application of MHT, as well as few studies using models of intracerebral glioblastoma.^(^ ^8^ ^,^ ^13^ ^)^ One of the magnetic nanoparticles that presents with a great potential is SPIONs coated with aminosilane (SPIONs_Amin_), as they present with a greater value of saturation magnetization (790.93 A/m) and influences the heating capacity, in comparison with SPIONs coated with other materials, for instance, carboxymethyl-dextran (227.13 A/m).^(^ ^15^ ^)^ Using SPIONs_Amin_ in assays with MHT, Jordan et al.,^(^ ^9^ ^)^ showed that SPIONs enabled the formation of more stable deposits of nanoparticles around the entire tumor, relative to SPIONs coated with dextran. Additionally, these SPIONs_Amin_ were evaluated and used for clinical applications in patients with tumors,^(^ ^16^ ^)^ and did not harm cortical neurons in animal model studies.^(^ ^17^ ^)^

In this sense, the present study seeks to elucidate, *in vitro* and *in vivo,* the properties of SPIONs coated with aminosilane associated with different parameters of MHT in the therapeutic efficiency of GBM in preclinical models. Therefore, we evaluated the characteristics of the appropriate AMF for MHT applied in the treatment of GBM tumors, due to the efficiency in heating of the SPIONs_Amin_, resulting from the intensity of the magnetic field and the frequency of oscillation, as well as the assessment of the efficacy of MHT therapy *in vitro* and *in vivo*, by means of the bioluminescence (BLM) technique.

## OBJECTIVE

To evaluate the potential of the magnetic hyperthermia using nanoparticles of iron oxide coated with aminosilane in models of glioblastoma tumors.

## METHODS

### Experimental design

**Figure 1:**
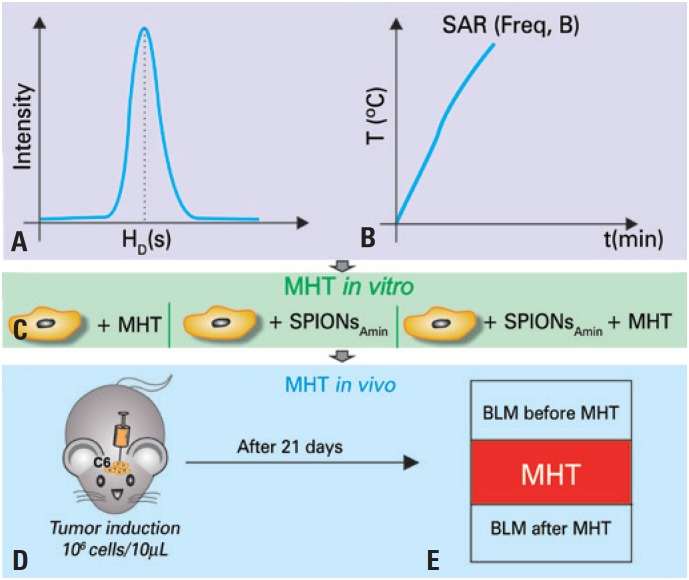
Stages of the experimental design. (A-B) The first stage covered the evaluation of stability of the hydrodynamic diameter [(H_D_(t)] and of the potential transformation of magnetic into thermal energy of the SPIONs coated by aminosilane (SPIONs_Amin_), obtaining the specific absorption rates (SAR) relative to the oscillation frequency (Freq) and intensity of the magnetic field (B). (C) In the second stage, the *in vitro* studies analyzed the viability of C6 cells with and without the magnetic hyperthermia application, as well as in the presence and absence of SPIONs_Amin_. (D) In the third stage, the glioblastoma tumor was induced (intracerebral application of 10^6^ C6 cells through stereotaxis), (E) for posterior application of magnetic hyperthermia 21 days after tumor induction, and evaluation of the efficacy of the therapy with the bioluminescence technique (before and after MHT) T: temperature; t: time: min: minutes; B: magnetic filed intensity; Amin: aminosilane; BLM: bioluminescence.

This study was developed in three stages. In the first stage, tests were performed of stability of the hydrodynamic size of the SPIONs_Amin_ (Figure 1A), as well as of the transformation potential of magnetic into thermal energy in the SPIONs_Amin,_ using different frequencies and intensities of magnetic field, obtaining, from the heating curves, the specific absorption rate (SAR) (Figure 1B). In the second stage, *in vitro* tests were performed to verify the viability of C6 cells with and without the application of MHT, in the presence and absence of SPIONs_Amin_ (Figure 1C). In the third stage, *in vivo* MHT assays were conducted, initiating with a tumor induction of intracerebral GBM in rats, by means of the application of 10^6^ C6 cells through stereotaxis (Figure 1D), followed by the tumor evaluation by BLM, pre- and post-application of MHT (Figure 1E), which used the parameters established in the first and second stages.

### Magnetic nanoparticles

SPIONs_Amin_ with a magnetite nucleus (Fe_3_O_4_) (Chemicell, Berlin, Germany), with a density of ∼1.25g/cm^3^, coupled with the functional amine (NH_2_) group, and HD 100nm and zeta potential +20mV.

### Size distribution of aminosilane-coated superparamagnetic iron oxide nanoparticles

To evaluate the polydispersion of the HD and stability of SPIONs_Amin_, the dynamic light scattering (DLS) technique was used, with the Zetasizer Nano S (Malvern, UK) system. The assessment was made at the concentration of 100mgFe/mL, and data were collected at 25°C, for a 60-second equilibrium period, with recordings every 30 minutes for 24 hours.

### Heating potential of the aminosilane-coated superparamagnetic iron oxide nanoparticles in the magnetic hyperthermia system

To determine the heating potential of SPIONs_amin_ with the MHT technique, an AMF system model DM100 (nB nanoScale Biomagnetics) was used, combining four frequencies (309, 557, 715, and 874kHz), and five intensities of magnetic field (100, 125, 150, 175, and 200 Gauss) to obtain the heating curves.

We used 100μL of SPIONs_Amin_ at the concentration of 5mg/mL dispersed in an aqueous medium, allocated in a thermally isolated recipient. The temperature of the SPIONs_Amin_ was monitored through an optical fiber system (Luxtron 3204), initiating all the analyses at 19°C.

The heating curves obtained were used to calculate the values of SAR in W/g for each frequency and magnetic field applied, according to equation 1:

(Equation 1) $$SAR=H_{water}\frac{\text{ΔT}/\text{δt}}{C_{Fe}}$$

where H_water_ is the specific heat of the water, ΔT/δt is the initial inclination of the temperature in °C/s, and C_Fe_ is the concentration of iron in g/mL.

### *In vitro* study

#### C6 cell culture

The C6 cell line was cultivated in RPMI medium (GIBCO^®^ Invitrogen Corporation, CA, USA), supplemented with 10% bovine fetal serum (BFS) (GIBCO^®^ Invitrogen Corporation, CA, USA), and incubated at 37°C (5% of CO_2_) until reaching a confluence of 70%, when it then was trypsinized to be used in the proposed trials.

The C6 cells were transduced with a luciferase lentiviral vector as per a previously established protocol,^(^ ^1^ ^8)^ resulting in the acquisition of cells transfected with luciferase for the evaluation of treatment with the BLM technique.

### Evaluation of magnetic hyperthermia *in vitro*

Samples containing 10^6^ C6 cells suspended in 200µL of RPMI 10% BFS were evaluated in triplicate under four conditions: C6 cells (Control Group), C6 cells marked with SPIONs_Amin_, C6 cells submitted to MHT, and C6 cells marked with SPIONs_Amin_ submitted to MHT. Marking of the C6 with SPIONs_Amin_ was performed with 600mg Fe/mL for 18 hours. In the samples submitted to MHT, we used the parameters of 200 Gauss and 874kHz for a period of 40 minutes. Efficiency of the MHT technique (cell viability) was evaluated with BLM, in which 100µL of luciferin were added to each sample, along with the posterior acquisition of images employing the IVIS Lumina III (XenogenCorp, USA) equipment.

### Study *in vivo*

#### Animals

Ten male Wistar rats at 2 months of age (weighing 290 to 350g) were used. The animals were acclimated at the Surgical Experimentation and Training Center (CETEC) of the *Instituto de Ensino e Pesquisa Albert Einstein* (CEUA 3126-17). This vivarium is accredited by the Association for Assessment and Accreditation of Laboratory Animal Care International (AAALAC). The animals were kept in individual boxes lined with autoclaved sawdust, with water and balanced feed *ad libitum,* following a light-dark cycle of 12 hours (7 am to 7 pm), with a temperature of 21°C (±2°C), as per international specifications.

### Glioblastoma tumor induction with C6 cells

The animals were anesthetized with ketamine (90mg/kg)and xylazine (12mg/kg). Implantation of C6 cells (10^6^/10µL of culture medium) was done by stereotaxis according to the established protocol.^(^ ^6^ ^)^ The point of implantation of cells in the right frontal cortex was determined and marked on the bone surface following orientations of Swansen's stereotaxical atlas:^(^ ^19^ ^)^ anteroposterior = 2.0mm; latero-lateral = 2.0mm; depth = 2.5mm.

### Magnetic hyperthermia process assay in the animal model

The *in vivo* MHT assays were performed 21 days after tumor induction. This application of tumor staging is based on previous studies of the group.^(^ ^20^ ^,^ ^21^ ^)^

Before being submitted to the MHT process, the control tumor evaluation was performed on the 21^st^ day with the BLM technique, using IVIS equipment in which the animals were maintained with inhaled anesthesia of isofluorane (2% saturation) with a flow of 2.5L/min of oxygen. The BLM signal was acquired after 5 minutes of administration of 100μL luciferin solution (1mM in PBS).

On the 22^nd^ day after tumor induction, 50µg of iron were applied contained in 10µL of the colloidal suspension of SPIONs_Amin_, divided into four equidistant points, 3mm from the central region of the tumor mass using stereotaxis equipment. After 20 minutes of the SPIONs_Amin_ application_,_ the animals were positioned inside the coil of the MHT equipment and submitted to the planned heating, composed of two parts. During the first part, the frequency of 874kHz and a field of 200 Gauss were used until reaching 42°C; during the second part, the frequency was maintained and the intensity of the field was modulated to maintain the temperature at 42°C, totaling up a period de 40 minutes of MHT. Mapping of the intratumor temperature was measured with an optical fiber (Luxtron 3204) with temperature probes (diameter of 0.55mm).

After MHT application, the animals were reassessed by means of BLM, and thus was determined the difference of the signal between the images before and after therapy in photons/second.

## RESULTS

### Dynamic light scattering

**Figure 2:**
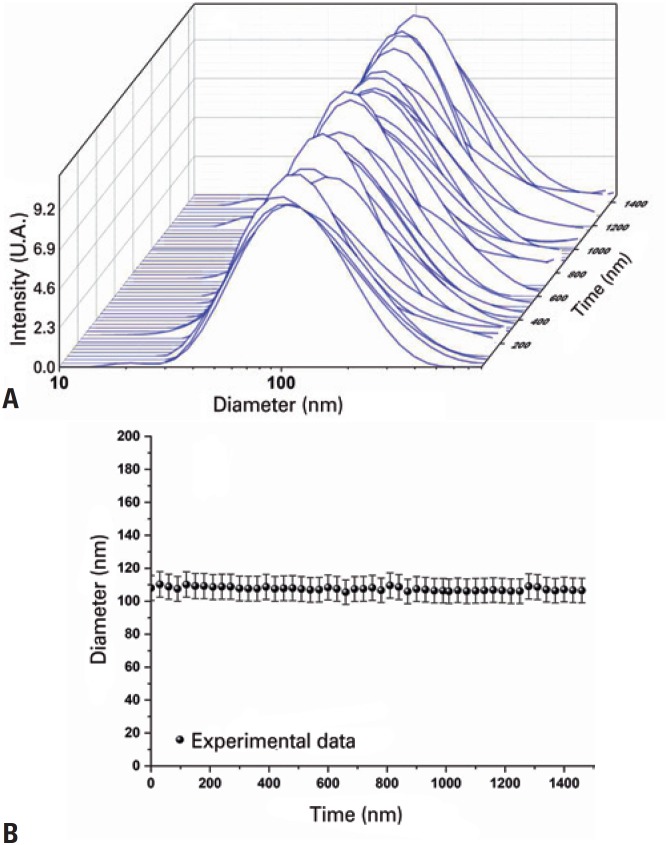
Stability of aminosilane-coated superparamagnetic iron oxide nanoparticles. (A) Polydispersion of the hydrodynamic diameter of the aminosilane-coated superparamagnetic iron oxide nanoparticles during a 24-hour period. (B) Values of the mean hydrodynamic diameter of the aminosilane-coated superparamagnetic iron oxide nanoparticles, with no significant difference (p>0.05) AU: arbitrary units.

The measurements of stability performed with the DLS technique on the SPIONs_Amin_ dispersed in water are shown in figure 2. The curves of figure 2A indicate that the SPIONs_Amin_ are polydispersed in the hydrodynamic size, in which the peak of maximal intensity corresponding to the mean HD was 110±5nm, with no significant difference between the measurements (p>0.05) acquired in 24 hours. Therefore, we can consider SPIONs_Amin_ stable during the period analyzed, as shown in figure 2B.

### Heating capacity of the aminosilane-coated superparamagnetic iron oxide nanoparticles

**Figure 3:**
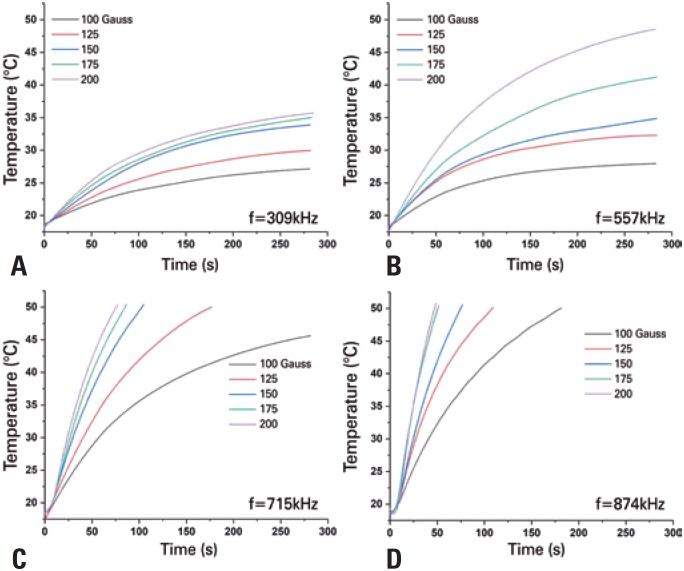
Heating curves of aminosilane-coated superparamagnetic iron oxide nanoparticles showing increment of the temperature as a function of time for samples submitted to intensities of alternating magnetic fields of 100, 125, 150, 175, and 200 Gauss, and frequencies of (A) 309kHz, (B) 557kHz, (C) 715kHz, and (D) 874kHz

The heating curves of the SPIONs_Amin_ obtained when submitted to AMF are shown in figure 3. The evaluation of heating shows that for samples submitted to AMD intensities varying from 100 to 200 Gauss for fixed frequencies, the heating temperature is incremented with the increase of the magnetic field (Figure 3A-D), as well as with the elevation of frequencies. Heating time also decreases with the use of elevated frequencies and AMF (Figure 3D). Aminosilane-coated superparamagnetic iron oxide nanoparticles dispersed in an aqueous medium under the action of an ADF of 200 Gauss and 874kHz, provided greater heating relative to other parameters, and the temperature reached 50°C in less than 50 seconds (Figure 3D).

### Specific absorption rate calculation

**Figure 4:**
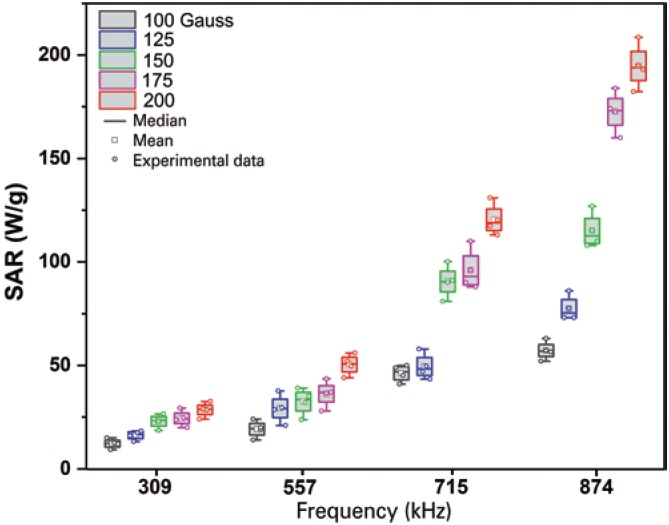
Specific absorption rate of the aminosilane-coated superparamagnetic iron oxide nanoparticles submitted to alternating magnetic field intensities of 100 (black), 125 (blue), 150 (green), 175 (pink), and 200 Gauss (red) for frequencies of 309, 557, 715, and 874kHz SAR: specific absorption rate.

The analysis of SAR (Figure 4) was carried out based on the heating curves previously obtained (Figure 3). It was noted that the exposure of SPIONs_Amin_ at 874kHz and 200 Gauss afforded a higher SAR (194.917W/g) among all the analyses (Figure 4). Table 1 shows the values of SAR in each analysis applied, and SAR depended on the intensity of the AMF and the frequency. The value of SAR measured at 200 Gauss within the frequency range of 309 to 874kHz is incremented by 28.5 to 194.7W/g, and is significant (p<0.001) relative to the frequencies analyzed, as well as in the fields analyzed (p<0.001), since in the case of SAR measured as 874kHz in the magnetic field of 100-200 Gauss there is an increment of 57.2 to 194.7W/g. Therefore, the values of SAR increased insofar as the established parameters of frequency and intensity of the magnetic field also raise; the results presented of the interaction between both the parameters are significant (p<0.001).

**Table 1:** Specific absorption rate values calculated for aminosilane-coated superparamagnetic iron oxide nanoparticles, under the effect of alternating magnetic fields of 100, 125, 150, 175, and 200 Gauss for 309, 557, 715, and 874kHz

Frequency of AMF(kHz)	Intensity of AMF (Gauss)	SAR (W/g)
309	100	12.2±2.4
	125	16.2±2.2
	150	23.0±3.4
	175	24.4±3.9
	200	28.5±3.5
557	100	19.2±4.1
	125	29.2±6.8
	150	32.5±6.4
	175	36.3±6.4
	200	50.4±5.0
715	100	46.2±4.1
	125	49.4±6.2
	150	90.6±7.9
	175	96.0±9.9
	200	120.4±7.7
874	100	57.2±4.5
	125	77.4±6.1
	150	115.1±8.5
	175	172.6±9.8
	200	194.7±10.8

By the variance analysis (ANOVA), a significant difference was verified among the fields and frequencies, and in the interaction between fields and frequencies with p<0.001. The data were previously tested as to distribution. AMF: alternating magnetic field; SAR: specific absorption rate.

### Evaluation *in vitro* of magnetic hyperthermia

**Figure 5:**
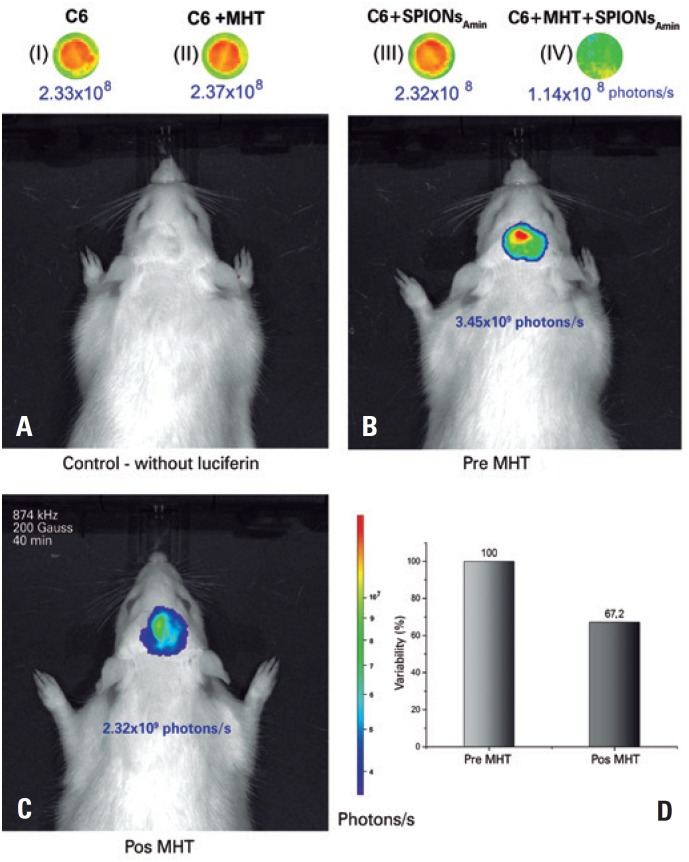
Evaluation of the magnetic hyperthermia (MHT). *In vitro* (I-IV assays were conducted, comparing to C6 cells alone (I), C6 + MHT (II), and C6 + aminosilane-coated superparamagnetic iron oxide nanoparticles (SPIONs_Amin_) (III), and C6 + SPIONs_Amin_ + MHT (IV), showed a 52% reduction in the intensity of bioluminescence in C6 cells marked with SPIONs_Amin_, and submitted to MHT. There was no significant difference between conditions (I), (II), and (III). *In vivo assays* (A-D) were performed, where in (A), we noted that bioluminescence in and of itself, without the application of luciferin, does not interfere in the capture of the bioluminescence signal. In (B), the tumor that developed 21 days after the tumor induction issued a bioluminescence relative to which, after (C) the application of MHT (200 Gauss, 874kHz) for 40 minutes, (D) a reduction of 32.8% was observed

The C6 cells of the control group, when observed by the BLM image, presented with an intensity of 2.33x10^8^ photons/s (Figure 5I), with no significant difference (p>0.05) as to the intensity of BLM of the C6 cells submitted to MHT (200 Gauss and 874kHz) in the absence of SPIONs_Amin_ (2.37x10^8^ photons/s) (Figure 5II) as to the intensity of BLM of the C6 cells in the presence only of SPIONs_Amin_ (2.32x10^8^ photons/s) (Figure 5III). These results indicate that neither AMF nor SPIONs_Amin_ by themselves interfere in the viability of C6 cells. However, in C6 cells that received MHT applications in the presence of SPIONs_Amin_, a 52% reduction was noted in cellular viability when compared to the controls (1.14x10^8^ photons/s) (Figure 5-IV).

### Evaluation of magnetic hyperthermia in the animal model by bioluminescence

On the 21^st^ day after tumor induction, the baseline evaluation of the tumor by BLM without the application of luciferin showed that the BLM equipment alone is not capable of biasing the analysis result (Figure 5A). After application of luciferin and before the application of the AMF, the tumor signal recorded by BLM was 3.45x10^9^ photons/s (Figure 5B). After 24 hours, MHT was applied (200 Gauss and 874kHz) for 40 minutes, with an observed signal decay to 2.32x10^9^ photons/s (Figure 5C). After application of MHT using the best conditions of AMF intensity and frequency previously tested *in vitro*, the decrease in tumor mass relative to the controls was 32.8% (Figure 5D).

## DISCUSSION

The use of nanotechnological resources applied to therapeutic techniques for aggressive tumors, such as the GBM, has represented an important role in preclinical studies, especially the use of magnetic nanoparticles for diagnostic and therapeutic purposes.^(^ ^22^ ^)^Superparamagnetic iron oxide nanoparticles have different physical and chemical characteristics with different biological interactions,^(^ ^23^ ^)^ such as the HD, in which the conservation of the size over time avoids the formation of clusters, making the SPIONs stabler,^(^ ^24^ ^)^ and consequently, more effective in the MHT technique.^(^ ^25^ ^)^ This is an important characteristic, albeit poorly explored relative to the description of stability of SPIONs. In the present study, the kinetics of the aggregation process of the SPIONs_Amin_ monitored for 24 hours by DLS, showed that there was no formation of clusters, maintaining the HD and its distribution polydispersed, a characteristic of the magnetic nanoparticles.^(^ ^26^ ^)^

After guaranteeing the stability of the SPIONs_Amin,_ the heating potential measured by SAR was evaluated with the objective of planning the therapeutic temperature. In the process of characterization of the heating curves obtained by the variations of amplitude and frequency of AMF, a forced hysteresis is evident in the SPIONs_Amin_, which contributes to the complex magnetization in heating, as well as the electromagnetic and thermal processes that influence heating.^(^ ^27^ ^)^ Heating of the aqueous dispersion of SPIONs_Amin_ when submitted to MHT essays, occurs due to a spatial arrangement of the SPIONs_Amin_, which under the action of the magnetic field, produces changes in the interparticulate dipole-dipole interactions and in the Brownian movement.^(^ ^28^ ^)^ A review study showed that magnetite nanoparticles with similar properties applied in preclinical studies, showed SAR values in the range of 96 to 286W/g.^(^ ^13^ ^)^ In clinical applications, SAR values are within the range of 0.12 to 2,452W/g, depending on the characteristics of the nanoparticles, as well as of the experimental parameters.^(^ ^25^ ^,^ ^29^ ^,^ ^30^ ^)^ Thus, the values of SAR found in our study were consistent with literature, in which the highest value of SAR was 194.917W/g for the colloidal suspension of the SPIONs_Amin_ submitted to AMF of 874kHz and 200 Gauss, proving adequate for the *in vivo* application of the MHT therapy.

Before the application of MHT *in vivo*, it was noted, through the BLM images in the *in vitro* sample, that AMF did not influence the viability of the C6 cells in the absence of SPIONs_Amin_, or the viability of the cells in the presence of SPIONs_AMIN_ in AMF. The BLM technique, due to its sensitivity, is used in the evaluation of tumor growth and of possible metastases,^(^ ^31^ ^)^ and has a good correlation with other imaging techniques, such as magnetic resonance and histology^(^ ^27^ ^,^ ^31^ ^)^ in monitoring of tumor growth.

The efficiency of MHT of the SPIONs_Amin_ administered locally in the tumor mass can be influenced by several factors, such as the dispersion of SPIONs_Amin_ throughout the tissues surrounding the administration site, which affects the heating produced by the SPIONs_Amin_ in the presence of AMF. Additionally, heating can be influenced by physiological aspects that are implicit in the calculation of heat transfer (Pennes equation), such as local metabolism, blood perfusion rates, coefficient of heat transfer in the tissues involved, specific mass of the blood, among others.^(^ ^32^ ^,^ ^33^ ^)^ In our study, we obtained 32.5% efficiency with a single application of MHT, using the lowest mass value of SPIONs_Amin_ (50µg Fe) in comparison with other studies that applied in the range of 0.5 to 3mg Fe in glioma tumors induced in the flank or subcutaneous regions.^(^ ^13^ ^)^ Use of high concentrations of iron nanoparticles can cause toxicity to neighboring tissues, as well as affect other organs associated in the biodistribution and elimination of nanoparticles through the liver, kidneys, and spleen.^(^ ^34^ ^)^

As a limitation of the study, we can point out that the efficiency of MHT through the evaluation of cell viability was not tested with a shorter AMF application time, or in its effect over time.

Preclinical studies of MHT showed promising results when combined with another treatment, such as radiation therapy or chemotherapy.^(^ ^35^ ^)^ Nonetheless, there are challenges to be overcome in order to increase the efficiency of MHT for clinical application. The increase in efficiency of MHT therapy depends of the prior determination of AMF parameters, which produce the adequate value of nanomaterial SAR, as well as the appropriate evaluation of *in vitro* and *in vivo* with molecular imaging techniques, such as BLM. Finally, we can affirm that SPIONs_Amin_ have the potential for MHT therapy in the treatment of GBM tumors.

## CONCLUSION

The present study evaluated the potential of magnetic hyperthermia using the aminosilane-coated superparamagnetic iron oxide nanoparticles for the treatment of glioblastoma in the animal model. The value of the specific absorption rate characterized for aminosilane-coated superparamagnetic iron oxide nanoparticles submitted to the action of an alternating magnetic field, indicates a good capacity for heating in order to reach the temperature. The bioluminescence technique showed, after application of magnetic hyperthermia therapy, a reduction in cell viability by 32.8% in the *in vitro* study and of 52% in the *in vivo* study. Therefore, application of magnetic hyperthermia proved to be promising for the therapeutic process of glioblastoma tumors using aminosilane-coated superparamagnetic iron oxide nanoparticles, which demonstrates favorable magnetic characteristics for the process of magnetic hyperthermia. Nevertheless, a careful assessment of other parameters involved, such as magnetic properties, polydispersion of superparamagnetic iron oxide nanoparticles, morphology, dose applied of superparamagnetic iron oxide nanoparticles, site of administration, periodic applications of the treatment, among others is necessary.
